# ChatGPT-Assisted Deep Learning Models for Influenza-Like Illness Prediction in Mainland China: Time Series Analysis

**DOI:** 10.2196/74423

**Published:** 2025-06-27

**Authors:** Weihong Huang, Wudi Wei, Xiaotao He, Baili Zhan, Xiaoting Xie, Meng Zhang, Shiyi Lai, Zongxiang Yuan, Jingzhen Lai, Rongfeng Chen, Junjun Jiang, Li Ye, Hao Liang

**Affiliations:** 1 Guangxi Key Laboratory of AIDS Prevention and Treatment & School of Public Health, Guangxi Medical University, Nanning, Guangxi, China; 2 Joint Laboratory for Emerging Infectious Diseases in China (Guangxi)-Association of Southeast Asian Nations, Life Sciences Institute, Guangxi Medical University, 22 Shuangyong Road, Qingxiu District, Nanning, Guangxi, 530021, China, 86 0771-5334215; 3 Life Sciences Institute, Guangxi Key Laboratory of AIDS Prevention and Treatment & Joint Laboratory for Emerging Infectious Diseases in China (Guangxi)-Association of Southeast Asian Nations, Guangxi Medical University, Nanning, China

**Keywords:** time series analysis, epidemic forecasting, public health preparedness, model optimization, seasonal pattern

## Abstract

**Background:**

Influenza in mainland China results in a large number of outpatient and emergency visits related to influenza-like illness (ILI) annually. While deep learning models show promise for improving influenza forecasting, their technical complexity remains a barrier to practical implementation. Large language models, such as ChatGPT, offer the potential to reduce these barriers by supporting automated code generation, debugging, and model optimization.

**Objective:**

This study aimed to evaluate the predictive performance of several deep learning models for ILI positive rates in mainland China and to explore the auxiliary role of ChatGPT-assisted development in facilitating model implementation.

**Methods:**

ILI positivity rate data spanning from 2014 to 2024 were obtained from the Chinese National Influenza Center (CNIC) database. In total, 5 deep learning architectures—long short-term memory (LSTM), neural basis expansion analysis for time series (N-BEATS), transformer, temporal fusion transformer (TFT), and time-series dense encoder (TiDE)—were developed using a ChatGPT-assisted workflow covering code generation, error debugging, and performance optimization. Models were trained on data from 2014 to 2023 and tested on holdout data from 2024 (weeks 1‐39). Performance was evaluated using mean squared error (MSE), mean absolute error (MAE), and mean absolute percentage error (MAPE).

**Results:**

ILI trends exhibited clear seasonal patterns with winter peaks and summer troughs, alongside marked fluctuations during the COVID-19 pandemic period (2020‐2022). All 5 deep learning models were successfully constructed, debugged, and optimized with the assistance of ChatGPT. Among the 5 models, TiDE achieved the best predictive performance nationally (MAE=5.551, MSE=43.976, MAPE=72.413%) and in the southern region (MAE=7.554, MSE=89.708, MAPE=74.475%). In the northern region, where forecasting proved more challenging, TiDE still performed best (MAE=4.131, MSE=28.922), although high percentage errors remained (MAPE>400%). N-BEATS demonstrated the second-best performance nationally (MAE=9.423) and showed greater stability in the north (MAE=6.325). In contrast, transformer and TFT consistently underperformed, with national MAE values of 10.613 and 12.538, respectively. TFT exhibited the highest deviation (national MAPE=169.29%). Extreme regional disparities were observed, particularly in northern China, where LSTM and TFT generated MAPE values exceeding 1918%, despite LSTM’s moderate performance in the south (MAE=9.460).

**Conclusions:**

Deep learning models, particularly TiDE, demonstrate strong potential for accurate ILI forecasting across diverse regions of China. Furthermore, large language models like ChatGPT can substantially enhance modeling efficiency and accessibility by assisting nontechnical users in model development. These findings support the integration of AI-assisted workflows into epidemic prediction systems as a scalable approach for improving public health preparedness.

## Introduction

In mainland China, influenza results in an annual average of 3 million excess outpatient and emergency visits attributable to influenza-like illness (ILI) [1]. Timely epidemic prediction plays a critical role in effective public health interventions. However, traditional surveillance systems often experience reporting delays, creating significant gaps in real-time outbreak response—an issue particularly challenging for frontline health workers tasked with rapid containment [2,3]. In recent years, deep learning models have shown considerable potential in time series analysis for disease trend forecasting. Architectures such as long short-term memory (LSTM) [4-9], neural basis expansion analysis for time series (N-BEATS) [10], temporal fusion transformer (TFT) [11], time-series dense encoder (TiDE) [12], and transformer [13] have demonstrated strong predictive performance across various epidemiological applications. Despite these advancements, the application of such models specifically to ILI forecasting remains relatively limited [14]. Current research in this area, including the studies of Darwish A et al [15], Yang et al [16], and Amendolara et al [17], has predominantly used LSTM-based approaches, while other advanced architectures have yet to be systematically explored for ILI prediction. Moreover, the practical implementation of deep learning methodologies presents a significant barrier. Building and optimizing such models requires specialized programming skills, which can limit their adoption by public health professionals, particularly those without technical backgrounds [18,19].

Addressing these practical barriers might be possible through the use of large language models (LLMs) such as ChatGPT [20,21], which have proven to be powerful tools in scientific research, natural language processing, and epidemic modeling. Built on advanced machine learning architectures, LLMs can support critical development tasks, including code generation, data preprocessing, error debugging, and model optimization [22]. In epidemiological modeling, this feature shows significant potential. Many frontline health practitioners and grassroots workers lack formal programming training, which limits their ability to independently apply deep learning models. LLMs provide a means to make advanced modeling techniques more accessible by aiding in technical implementation via natural language interactions. Although LLMs have been increasingly applied in fields such as healthcare [23] and medical diagnostics [24], their integration into influenza epidemic forecasting and early warning systems remains rare. Existing studies have largely focused on traditional model design and implementation, with limited attention to how LLMs can actively assist in the modeling workflow for infectious disease prediction.

In this study, we aimed to predict the ILI positivity rate of mainland China by implementing 5 deep learning architectures (LSTM, transformer, N-BEATS, TFT, and TiDE), thereby eliminating these gaps. Additionally, we investigate the auxiliary role of ChatGPT in supporting the model development process, particularly in tasks such as code generation, debugging, and performance optimization. In this way, we attempt to demonstrate that frontline health workers can bypass traditional programming barriers and run advanced predictive models through natural language interaction with LLM, thereby narrowing the gap between epidemiological research and primary public health practice.

## Methods

### Ethical Considerations

The data used in this study were sourced from a publicly accessible secondary database. Therefore, formal ethical approval was not required.

### Data Source

The data on the positive rate of ILI cases from the southern and northern regions of mainland China, as well as nationwide, spanning from the 1st week of 2014 to the 39th week of 2024, were obtained from the public database of the Chinese National Influenza Center (CNIC) [25].

### Data Preprocessing and Model Input

The dataset was divided into a training set and a validation set. Specifically, the training set comprised data from the 1st week of 2014 to the 52nd week of 2023, which was used to develop and train the models. The validation set covered data from the 1st week to the 39th week of 2024 and was used to evaluate the models’ predictive performance on unseen, future data. This chronological partitioning permitted evaluation of the models’ capacity to extrapolate from historical patterns to accurately forecast emerging ILI trends in real-world settings.

### Construction of LSTM Models

LSTM networks, a specific form of recurrent neural network, were used to capture temporal dependencies in the time series of ILI positive rates. By using memory cells with gating mechanisms, the LSTM design regulates information flow, which helps the model capture short-term fluctuations and long-term seasonal patterns crucial for predicting ILI. Through the use of input, forget, and output gates, LSTM dynamically processes raw high-dimensional ILI time series, integrating essential data into a fixed-size hidden state vector. The stacked layers use trainable nonlinear transformations to capture seasonal patterns and sudden changes, effectively reducing dimensions and extracting features within the network. The core computational mechanism of our LSTM implementation can be expressed as:

Forget gate:


$$f_{t}=\sigma (W_{f}x_{t}+U_{f}h_{t-1}+b_{f})$$

Input gate:


$$i_{t}=\sigma (W_{i}x_{t}+U_{i}h_{t-1}+b_{i})$$

Candidate cell state:


$${\tilde{c}}_{t}=tanh\left(W_{c}x_{t}+U_{c}h_{t-1}+b_{c}\right)$$

Cell state upstate:


$$c_{t}=f_{t}⊙c_{t-1}+i_{t}⊙{\tilde{c}}_{t}$$

Output gate:


$$o_{t}=\sigma (W_{o}x_{t}+U_{o}h_{t-1}+b_{o})$$

Hidden state:


$$h_{t}=o_{t}⊙tanh(c_{t})$$

In these equations, σ denotes the sigmoid activation function, tan h represents the hyperbolic tangent function, and indicates element-wise multiplication. The formulation helps the model to retain significant historical information and discard irrelevant noise, making it particularly effective for capturing the complex temporal patterns in ILIL epidemiological data. The LSTM networks were confirmed with bidirectional layers to process sequence information in both forward and backward directions, enhancing the model’s ability to detect contextual patterns in the time series.

### Construction of N-BEATS Models

To forecast ILI positive rates, the N-BEATS model was applied, taking advantage of its deep learning architecture to understand complex temporal trends in epidemiological information. The N-BEATS model uses a stack of fully connected neural network blocks, each made up of multiple layers with 2 residual connections: one dedicated to backcasting to reconstruct the input, and another for forecasting to predict future values. This design, featuring 2 layers of residuals, enables the model to incrementally refine its predictions by targeting the residuals not captured by earlier blocks, thereby improving forecast accuracy. The N-BEATS model uses each block to convert the input time series into basis expansion coefficients, which are subsequently combined with predefined basis functions to create the backcast and forecast outputs. Mathematically, the forecast ${\hat{y}}_{t+1:t+H}$ is obtained by summing the outputs of each stack:


$${\hat{y}}_{t+1:t+H}=\sum_{i=1}^{S}F_{i}\left(x_{t-L+1:t}\right)$$

where $F_{i}$ represents the forward projection function of the i-th stack,

and $x_{t-L+1:t}$ denotes the historical input sequence of length L.

### Construction of TFT Models

The TFT model was used to predict ILI positive rates, using its hybrid structure that merges recurrent neural networks with attention mechanisms to grasp both short-term and long-term patterns. TFT consists of several crucial parts: variable selection networks that dynamically select important input features at each time interval; static covariate encoders that manage time-invariant information like regional traits; and multi-head attention mechanisms that represent complex temporal interactions across multiple time scales. At the core of TFT’s design is the understandable multi-head attention mechanism, which calculates attention weights through the scaled dot-product formula:


$$Attention(Q,K,V)=softmax(\frac{QK^{T}}{\sqrt{d_{k}}})V$$

where Q, K, and V represent query, key, and value matrices derived from the input sequence, and $d_{k}$

is the dimension of the key vectors. By using this mechanism, the model can prioritize historical data based on its importance for future predictions, enhancing both accuracy and comprehensibility. Furthermore, TFT applies gating mechanisms and residual connections to support efficient information flow and alleviate issues such as vanishing gradients. By combining static metadata with temporal characteristics, TFT can address regional disparities in ILI transmission, making it highly effective for capturing the non-stationary and diverse nature of epidemiological time series data.

### Construction of TiDE Models

The TiDE model uses an encoder-decoder architecture that relies exclusively on multilayer perceptrons to effectively capture long-range dependencies and model non-stationary time series. In contrast to standard sequence models that use recurrence or convolution, TiDE simplifies forecasting by applying feedforward transformations to each time step separately while preserving temporal structure with positional and contextual encodings. With reduced computational complexity and the ability to support highly parallelized training, this design is apt for large-scale epidemiological forecasting tasks like ILI prediction. The core projection mechanism in TiDE can be expressed as:


$$Z_{t}=\phi (W_{z}X_{t}+b_{z})$$

where *Zt* represents the encoded state at time t, *ϕ* is a nonlinear activation function, *Wz* is the projection weight matrix, and *Xt* is the input vector of ILI features. The formulation supports the sharing of parameters over time steps, enabling the model to capture temporal dynamics through depth rather than through recurrence. Several stacked residual multilayer perceptron blocks form the encoder, enabling the transmission of information and the step-by-step abstraction of time-dependent patterns in the input sequence. TiDE also includes context gating, normalization, and skip connections to stabilize optimization and help model long-range patterns while maintaining local variability. Similarly, its decoder uses feedforward layers to transform the learned representations into forecasts over multiple steps.

### Construction of the Transformer Models

A self-attention-based encoder-decoder architecture was used in the transformer model to optimize sequential ILI data handling. Transformers, unlike recurrent networks, simultaneously process entire sequences with the help of positional encodings and multi-head attention. The model can grasp complex dependencies between observations at different times due to this design, no matter the temporal gap. At the core of the transformer’s function is the scaled dot-product attention mechanism, which is described as:


$$Attention(Q,K,V)=softmax(\frac{QK^{T}}{\sqrt{d_{k}}})V$$

where Q, K, and V represent query, key, and value matrices derived from the input sequence, and is the dimension of the key vectors. The softmax function balances attention weights throughout the sequence, facilitating the model’s ability to focus on crucial parts of the input during the generation of each output. The transformer’s self-attention mechanism allows the model to identify complex relationships between observations over different time intervals for ILI forecasting. This feature enables the model to concentrate on pertinent historical data points dynamically, no matter where they appear in the sequence. Furthermore, the transformer’s design allows for parallel processing of whole sequences, enabling efficient training and inference, which is a major benefit when dealing with large epidemiological datasets. The model’s proficiency in handling long-range dependencies without recurrent structures allows it to effectively capture the complex temporal dynamics found in ILI time series data. Previous research has shown these characteristics using transformer-based models for epidemiological forecasting.

### ChatGPT-Assisted Model Development

In this study, the ChatGPT large language model was used as an auxiliary tool to systematically support the development of deep learning models. This assistance primarily involved 2 key aspects: code generation and debugging support.

For code generation, structured prompt templates were designed to produce initial code frameworks for each model architecture. Standardized query formats were used (eg, "Generate a Python script for time-series prediction using the Darts library and the transformer model, including steps for data loading, preprocessing, model definition, training, prediction, and evaluation”) to ensure consistency and comprehensiveness across implementations. This approach facilitated efficient and reproducible development of complex model architectures without the need for extensive manual coding.

During debugging, code errors and related details were shared with ChatGPT for help. When working through data preprocessing steps, like transforming and scaling time series data, this approach was really useful for catching and fixing issues such as problems with converting dates or mismatched scales. We kept track of all error types and how we fixed them so we could create a clear debugging guide.

All interactions with ChatGPT followed specific prompt patterns to keep everything consistent and easy to reproduce. Example prompts are included in the extra materials in Multimedia Appendix 1. Using AI this way provides a repeatable method for building deep learning models in epidemiology, making it easier for more people to use predictive tools in public health without needing advanced technical skills.

### Cost Function and Parameter Learning Strategy

The mean squared error (MSE) was used as the loss function for training all deep learning models in this study, and it is defined as$MSE=\frac{1}{n}\sum_{i=1}^{n}(y_{i}-{\hat{y}}_{i}{)}^{2}$ , where ${\hat{y}}_{i}$ represents the predictive value and $y_{i}$ represents the actual value, and n is the total number of observations.

The model’s parameters were fine-tuned using gradient descent learning, particularly using the Adam optimizer alongside backpropagation. The learning rate and weight decay settings were adjusted manually for each architecture according to initial experiments, as follows:

TiDE: learning rate=0.0005, weight decay=1e-2

Transformer: learning rate=0.0001, weight decay=1e-3

N-BEATS: learning rate=0.0005, weight decay=1e-3

LSTM: learning rate=0.005, weight decay=1e-4

TFT: learning rate=0.001, weight decay=1e-3

In order to prevent overfitting and improve convergence, learning rate scheduling is implemented using the ReduceLROnPlateau strategy in all models. If the training loss does not improve within a patience window of 10 epochs, the learning rate will be reduced by 0.1. Dropout layers and batch normalization were applied to specific architecture design applications to enhance generalization.

### Software and Libraries

All models were implemented in Python using TensorFlow and Keras for LSTM, N-BEATS, TFT, and transformer models, with PyTorch used for some prototyping tasks. Time series forecasting and model evaluation were carried out using the Darts library. Data preprocessing and manipulation were done with NumPy and Pandas, while Matplotlib and Seaborn were used for visualization. SciPy was used for statistical analysis and hyperparameter optimization. Additionally, ChatGPT was used for code generation, debugging, and model optimization throughout the study.

### Model Comparison

To evaluate the performance of the models, we used several metrics: MSE, root mean squared error, mean absolute error (MAE), and mean absolute percentage error (MAPE). Smaller values of these metrics indicate superior predictive accuracy. The formulas for these metrics are as follows:


$$MSE=\frac{1}{n}\sum_{i=1}^{n}(y_{i}-{\hat{y}}_{i}{)}^{2}$$

$$MAE=\frac{1}{n}\sum_{i=1}^{n}|y_{i}-{\hat{y}}_{i}{|}^{2}$$

$$MAPE=\frac{100\%}{n}\sum_{i=1}^{n}|\frac{y_{i}-{\hat{y}}_{i}}{y_{i}}|$$

where ${\hat{y}}_{i}$ represents the predictive value and $y_{i}$ represents the actual value, and n is the total number of observations.

## Results

### Characteristics of Influenza-Like Symptoms in Mainland China

**Figure 1.**
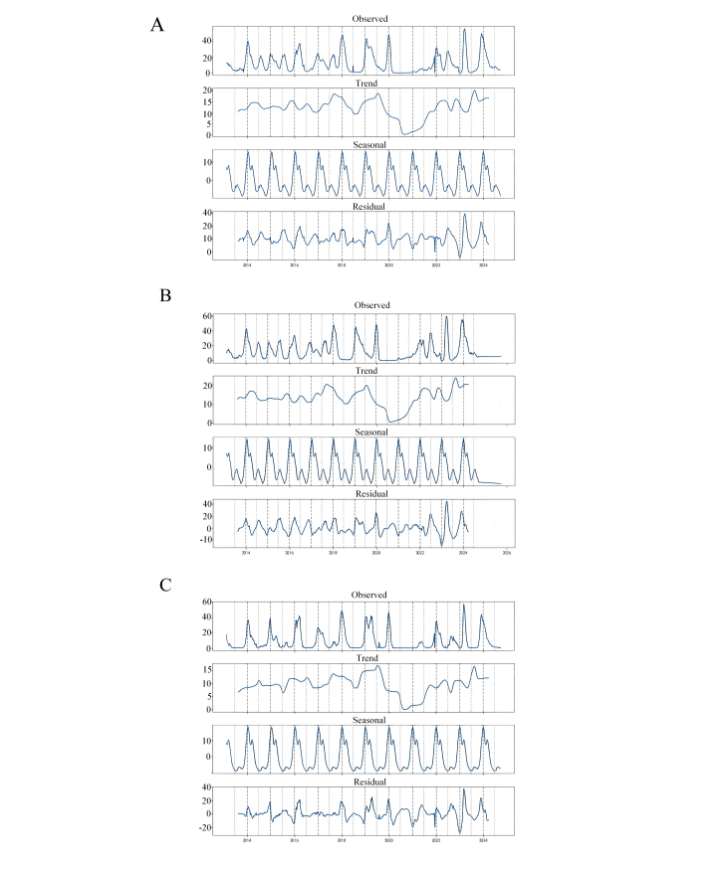
Seasonal decomposition of influenza-like illness (ILI) in mainland China. (A) Multicycle superposition of ILI cases at the national level; (B) seasonal variation patterns in the southern region; and (C) seasonal variation patterns in the northern region.

We systematically collected ILI case data from southern China, northern China, and mainland China as a whole between the 1st week of 2014 and the 39th week of 2024. Time-series analysis revealed consistent seasonal patterns across all regions, characterized by winter peaks (January-February) and summer troughs (July-August), as visually demonstrated in Figure 1A–C. The data revealed that the positive rate of ILI cases exhibited significant variability during 2014‐2024 (CV=0.99 nationwide, 0.96 in southern regions, and 1.32 in northern regions). Despite these cyclical patterns, the Augmented Dickey-Fuller test confirmed significant non-stationarity in the data, indicating inherent unpredictability in long-term trends.

A sharp decline in national ILI cases was observed in January 2020, coinciding with the introduction of COVID-19 containment measures such as social distancing and mask mandates. This trough was followed by a rebound to peak levels in January 2022 (Figure 1A). Regional analyses showed synchronized downward trajectories: southern China exhibited a sustained decline from July 2019 to July 2020 (Figure 1B), while northern China experienced a similar trend starting one month earlier (June 2019–July 2020, Figure 1C). Decomposition of the post-2020 period revealed dual dynamics, with overall case counts showing steady growth from late 2020, while positivity rates initially declined before rising again in early 2021.

Between 2014 and 2020, flu activity peaks stayed consistent during expected high seasons. Starting in 2020, we saw a 4‐6week delay in peak timing, likely connected to COVID-19-related behavior changes like mask use and social distancing. This shift reached its highest point in spring 2023, then returned to typical winter-summer patterns by 2024. By late 2023, all areas showed regular seasonal trends again, confirming stable disease spread patterns. These patterns reveal how flu transmission timing changes over years and help build better prediction models.

### Performance Comparison of Deep Learning Models

**Figure 2.**
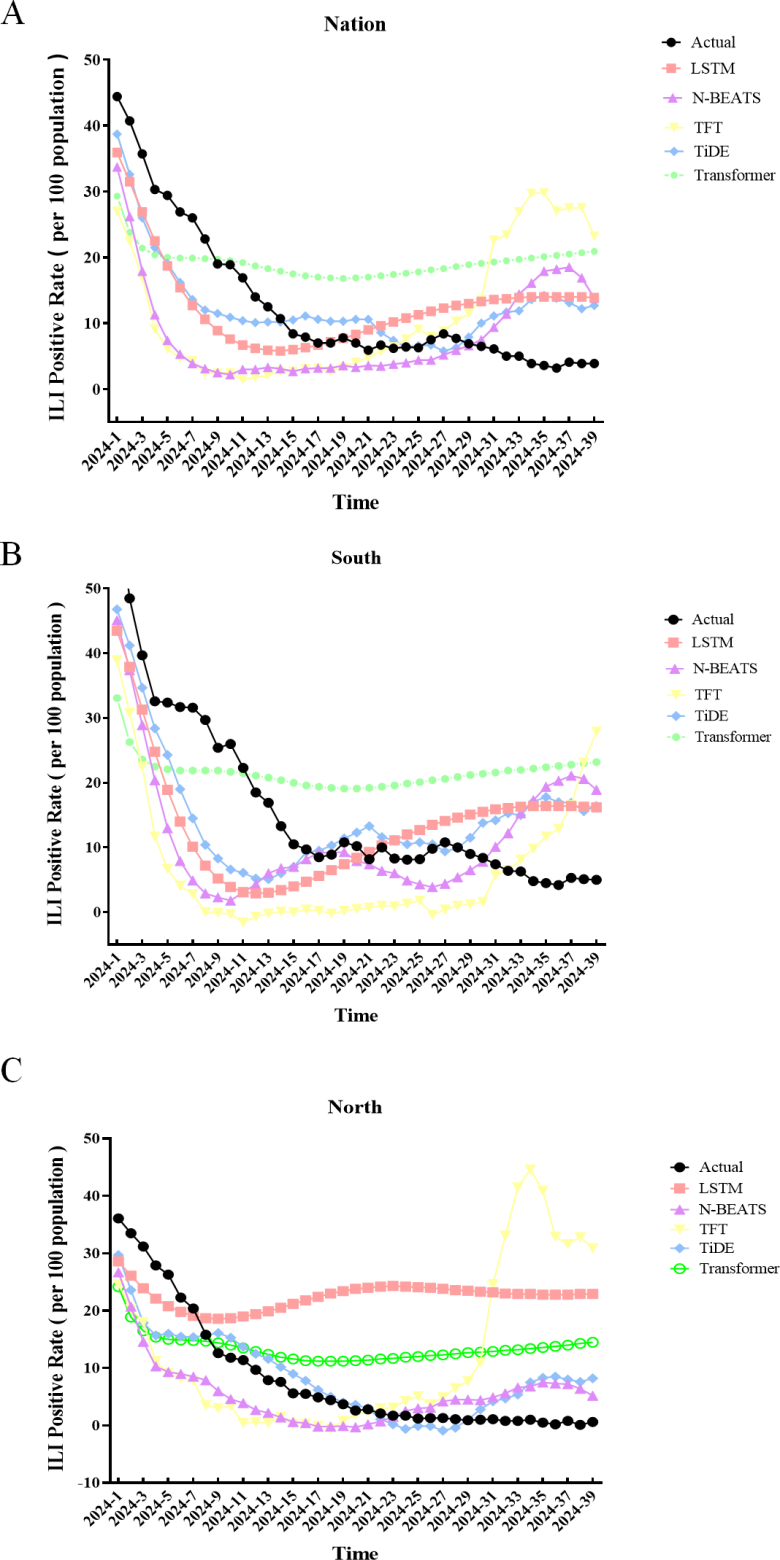
The comparative forecasting trajectories of multiple predictive models alongside actual observed data across 3 geographical divisions: Nation (**A**), South (**B**), and North (**C**). (from 2014 to 2023). “Actual” denotes the real data; LSTM represents the long short-term memory neural network model; N-BEATS (neural basis expansion analysis for time series), TFT (temporal fusion transformer), TiDE (time-series dense encoder), and transformer stand for distinct forecasting models. Specifically, the orange line indicates the actual data, the yellow line shows the predictive results from the LSTM model, the purple line represents predictions via the N-beats model, the green line reflects forecasts from the TFT model, the blue line is the predictive output of the TiDE model, and the pink line denotes predictions by the transformer model. ILI: influenza-like illness.

We evaluated the predictive performance of 5 deep learning models—LSTM, N-BEATS, TFT, TiDE, and transformer—in forecasting ILI positivity rates across national, southern, and northern datasets for weeks 1‐39 of 2024. Model training progress was monitored using fitting visualizations (Figure 2).

Among the 5 models, TiDE achieved the best overall performance, with predictions closely aligning with observed ILI-positive rates across all datasets. The models were assessed using 3 metrics: MSE, MAE, and MAPE, with lower values indicating better predictive accuracy.

**Figure 3.**
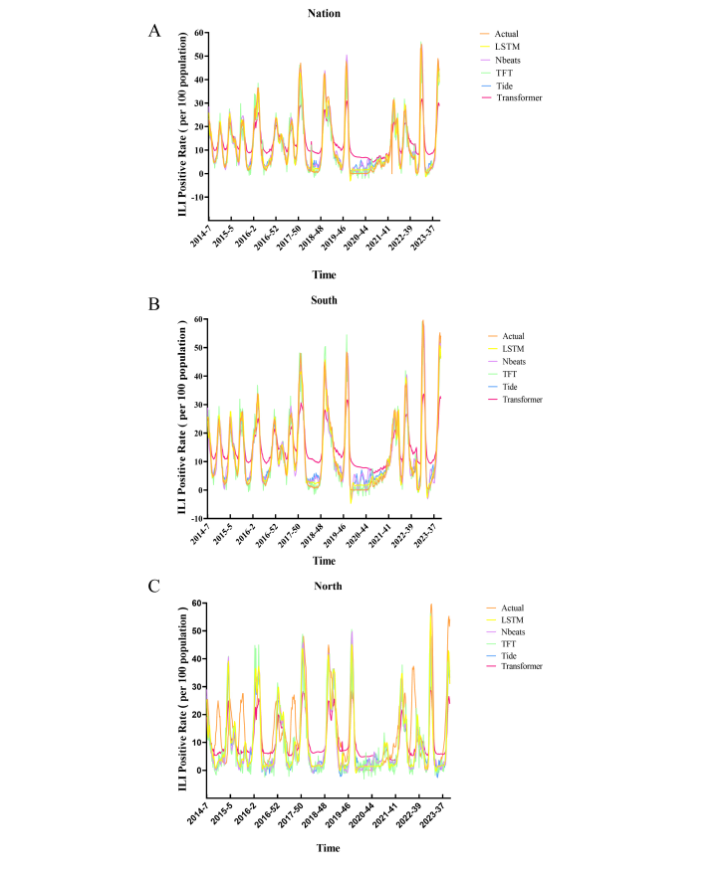
The forecasting curves of various models and the actual data series in 3 scenarios: Nation (A), South (B), and North (C). Comparison of different forecasting models. “Actual” denotes the real data; LSTM represents the long short-term memory neural network model; N-BEATS (neural basis expansion analysis for time series), TFT (temporal fusion transformer), TiDE (time-series dense encoder), and transformer stand for distinct forecasting models. Specifically, the black line indicates the actual data, the pink line shows the predictive results from the LSTM model, the purple line represents predictions via the N-BEATS model, the yellow line reflects forecasts from the TFT model, the blue line is the predictive output of the TiDE model, and the green line denotes predictions by the transformer model. ILI: influenza-like illness.

At the national level, TiDE performed better than the other models, with an MAE of 5.551, MSE of 43.976, and MAPE of 72.413%. N-BEATS was close behind, showing an MAE of 9.4231, MSE of 133.1737, and MAPE of 105.72%. LSTM performed moderately, with an MAE of 6.934, MSE of 61.391, and MAPE of 88.793%. Transformers and TFT had higher error rates, with transformers having an MAE of 10.6128 and TFT showing the largest deviation, with a MAPE of 169.29% (Figure 3A and Table 1).

In the southern region, TiDE again achieved the best performance (MAE=7.554, MSE=89.708, MAPE=74.475%). N-BEATS ranked second, while TFT and transformer displayed considerable deviations. LSTM performed well but remained slightly behind TiDE (Figure 3B, Table 1). In the southern region, TiDE achieved the highest performance once more (MAE=7.554, MSE=89.708, MAPE=74.475%). N-BEATS was the runner-up, and both TFT and transformer had significant deviations. LSTM did well but was marginally behind TiDE (Figure 3B and Table 1).

In the northern region, predictive performance varied significantly. TiDE maintained the lowest MAE (4.131) and MSE (28.922) despite high percentage errors (MAPE=486.087%). N-BEATS showed stable performance (MAE=6.325, MSE=58.0936, MAPE=468.41%). LSTM and TFT produced particularly high MAPE values (1918.52% and 2215.66%, respectively), while the transformer achieved intermediate error rates (MAPE=1090.63%) (Figure 3C and Table 1).

Overall, TiDE consistently achieved the highest accuracy and stability across regions, while notable forecasting challenges remained in the northern dataset due to higher variability and error amplification.

**Table 1.** Comparison of the fitting and prediction accuracy of the models.

			Training set			Test set	
Region	Model	MAE^a^	MSE^b^	MAPE^c^ (%)	MAE	MSE	MAPE (%)
	LSTM^d^	2.022	9.341	747.490	6.934	61.391	88.793
	N-BEATS^e^	2.468	11.296	1105.892	9.423	133.174	105.717
Nation	TFT^f^	2.290	12.059	564.371	12.538	235.157	169.290
	TiDE^g^	2.549	13.062	1338.139	5.551	43.976	72.413
	Transformer	5.658	47.188	3473.858	10.613	132.582	161.581
	LSTM	2.216	10.390	573.356	9.460	126.399	85.560
	N-BEATS	3.157	18.556	1079.920	9.898	160.962	88.502
South	TFT	2.134	9.599	328.874	13.385	243.345	106.853
	TiDE	2.876	15.516	972.010	7.554	89.708	74.475
	Transformer	6.266	57.100	2719.575	11.539	157.023	132.782
	LSTM	2.136	9.997	1156.550	16.119	314.581	1918.516
	N-BEATS	2.431	13.461	1740.311	6.325	58.094	468.412
North	TFT	2.629	14.188	1639.950	13.610	336.891	2215.656
	TiDE	2.701	15.989	2194.821	4.131	28.922	486.087
	Transformer	5.516	49.326	4291.066	9.267	101.251	1090.626

a MAE: mean absolute error.

b MSE: mean squared error.

c MAPE: mean absolute percentage error.

d LSTM: long short-term memory.

e N-BEATS: neural basis expansion analysis for time series.

f TFT: temporal fusion transformer.

g TiDE: time-series dense encoder

### Auxiliary Effects of ChatGPT in Model Development

ChatGPT was systematically integrated as an auxiliary tool throughout the model development process. Standardized prompt templates were designed to specify functional requirements, architectural details, and expected outputs. Code generated by ChatGPT required only minimal adjustments, mainly related to dataset-specific preprocessing and hyperparameter configuration. This approach was particularly effective for implementing complex models such as TiDE and N-BEATS, which would otherwise demand extensive manual coding.

**Figure 4.**
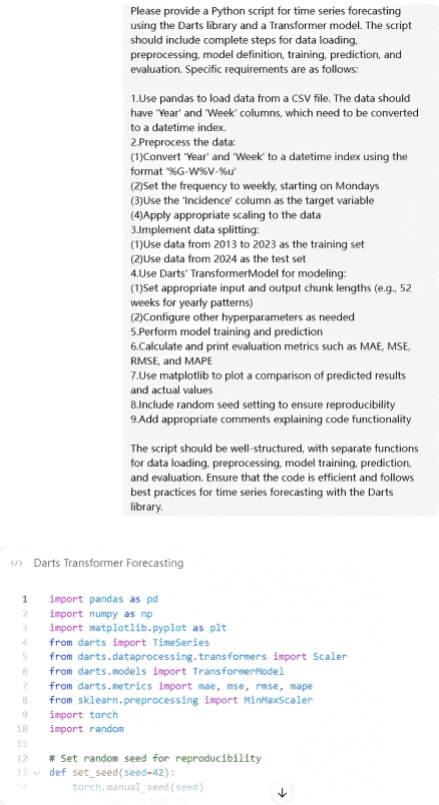
Demonstrating ChatGPT’s supporting role in model development: A case study on transformer code generation using prompt templates.

During the debugging process, a structured protocol was established, which recorded error messages, code context, and expected behavior for analysis by ChatGPT. For example, when resolving a syntax error related to type annotations (“illegal target for annotation”), ChatGPT accurately identified the source of the error as an illegal annotation on the right side of a variable assignment. This process significantly reduced debugging time and improved implementation efficiency. Similar benefits were observed in optimizing memory usage during the development of the transformer model; ChatGPT identified inefficient tensor operations causing computational bottlenecks (Figures4  5).

The results indicate that LLM-assisted workflows can help overcome technical barriers and improve the efficiency of developing deep learning–based epidemic prediction models.

**Figure 5.**
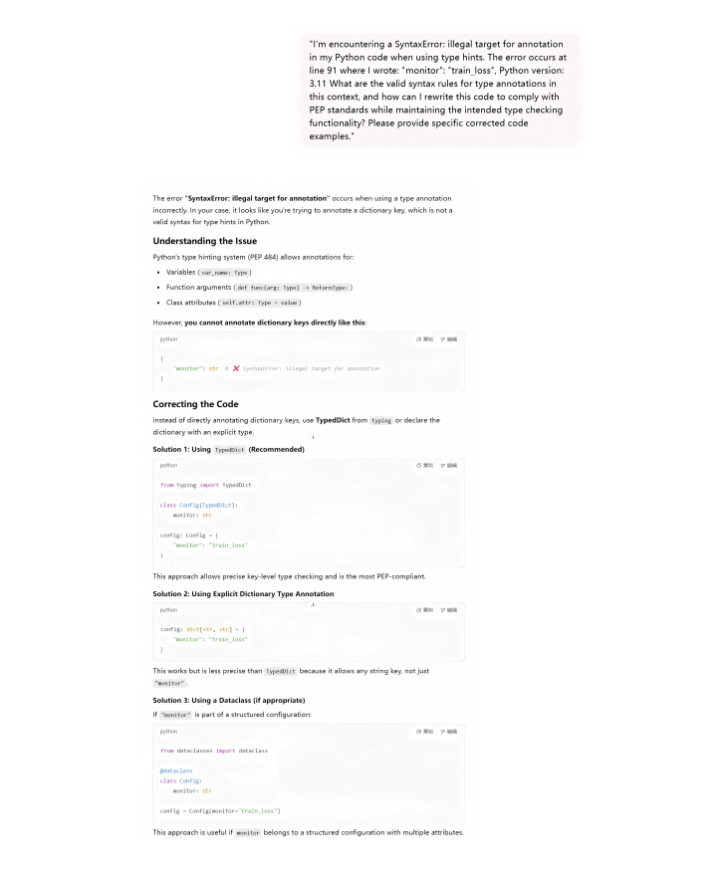
ChatGPT efficiently detects and corrects misdiagnoses, substantially reducing the duration of the debugging process.

## Discussion

### Overview

This study evaluated 5 deep learning models for predicting ILI rates across China while examining how ChatGPT supports predictive model creation. We compared the effectiveness of different AI architectures and implemented an organized AI-assisted development process. Our work resolves 2 key research gaps: limited testing of non-LSTM models and the absence of organized AI-supported approaches for flu prediction. The results advance practical modeling methods that health agencies can reliably apply for outbreak prediction. These outcomes highlight both the benefits and limitations of AI-powered disease forecasting tools, offering actionable guidance for improving public health monitoring systems and research methods.

The study used publicly available data on the positivity rate of ILI from the CNIC. The data are sourced from centralized weekly testing and reporting of clinical samples at national surveillance sites, having passed stringent quality control and outlier verification. For weeks with missing or delayed reports, we use linear interpolation to fill in the gaps and remove obviously abnormal samples to ensure the consistency and completeness of the data. The dataset includes data from the first week of 2014 to the 39th week of 2024, totaling over 10 years and about 545 consecutive time series samples, divided into national, southern, and northern levels to support analysis of regional differences. By analyzing long-term series data, it is clear that there are significant seasonal variations in Chinese mainland and ILI cases in the south and north (Figure 1). The high incidence in winter (January and February) and the low incidence in summer (July and August) are consistent with established epidemiology of influenza in temperate regions. The time series shows typical winter peaks and summer troughs; the seasonality aligns with previous studies by Ye et al [26], Chen et al [27], and Liu et al [28], who documented similar patterns in China populations. This seasonal variation is closely related to many factors and provides key information for the construction and prediction of subsequent models. It emphasizes the importance of choosing appropriate modeling methods that can adapt to observed changes.

Even though we see a clear seasonal pattern in the data, there’s still a lot of ups and downs. This variation likely comes from a mix of different factors. First, influenza viruses tend to change a lot—mutating and drifting so their spread isn’t always steady [29-31]. Second, the level of immunity in the population varies, especially as people lose immunity over time or get vaccinated each year differently [32]. In 2022, changes in COVID-19 policies, lifting restrictions, and shifting how health care resources are used also played a big role in how cases are reported [33]. Also, improvements in public habits, like wearing masks and people’s willingness to seek health care, have affected the data [34,35]. The pandemic also changed how people move around, which impacts how easily the virus spreads. All these factors together make the data unpredictable, making it tricky for models to predict accurately. They need to handle irregular patterns and unexpected changes.

On the validation set of weeks 1‐39 in 2024, the national MAE of the TiDE model was 5.55, MSE=43.98, and MAPE=72.41%; in the south, MAE=7.55, MSE=89.71, and MAPE=74.48%; in the north, MAE=4.13, MSE=28.92, and MAPE=486.09%. According to the results, the model effectively predicts outcomes in a variety of data and regions. The performance comparison of deep learning models highlighted significant regional variations in predictive ability. The encoder-decoder architecture of the TiDE model appears to more effectively understand the temporal complexities of ILI transmission patterns, as evidenced by its superior performance both nationally and in southern China. Yet, the considerably poorer results of all models in northern China, even the TiDE model, which fares relatively better, point to core challenges in modeling the ILI dynamics of this region. The regional differences are probably due to the more extreme seasonal temperature changes in northern China, variations in population density, and possibly greater inconsistencies in data from surveillance reporting systems. The introduction of these factors creates nonlinear complexities that current deep learning frameworks have difficulty fully understanding, pointing to the necessity for region-specific modeling techniques. The use of MSE as the loss function, along with the Adam optimizer and learning rate scheduling, also contributed to stable training and convergence across all model architectures, ensuring an equitable comparison of predictive performance.

Using ChatGPT as a helpful research tool offers a better way to build disease prediction models. Unlike older methods that demand strong coding skills, this AI-supported approach makes advanced analysis easier to use through ready-to-use question templates and error-checking steps. Importantly, ChatGPT wasn’t part of the final prediction system—it only helped create and improve the code. While AI tools have been tested for helping doctors make decisions, using them to build disease spread models is still new. This method allowed testing multiple AI models (LSTM, N-BEATS, TFT, TiDE, and transformer) side-by-side using the same basic setup.

### Limitations

This study has several limitations. First, we did not establish a comprehensive systematic uncertainty quantification framework—encompassing intrinsic model stochasticity, data reporting delays, policy-induced systemic variations, and parametric or nonparametric uncertainties—which would require dedicated computational infrastructure. Specifically, internal uncertainties originate from inherent model randomness (eg, parameter initialization and stochastic dropout mechanisms), external uncertainties relate to data latency, policy adjustments, and demographic fluctuations, while structural uncertainties reflect unmodeled latent factors in influenza transmission dynamics. Current LLMs (eg, ChatGPT) lack the mathematical rigor necessary to support such complex probabilistic modeling [36,37] .

Second, we could not precisely quantify potential uncertainty sources during model development due to incomplete standardization of data logging. This study prioritized validating framework feasibility over achieving precise estimation of specific uncertainty parameters. We therefore recommend subsequent investigations using enhanced quantitative validation tools. The present work provides a qualitative analytical framework, suggesting future research should focus on 2 empirically supported directions: developing LLM-compatible uncertainty quantification modules and validating time-dependent impacts of uncertainty factors on predictive performance through longitudinal studies, approaches that have demonstrated feasibility in analogous infectious disease modeling research.

### Conclusions

Deep learning models could significantly improve early disease warning systems by accurately predicting flu-like outbreaks (ILI). Using ChatGPT-supported systems to automatically generate code, fix errors in real-time, and improve prediction models allows researchers to follow standardized procedures—reducing technical skill demands and speeding up studies. However, these models need to prove they work reliably in different regions, especially in northern China’s complex disease spread patterns, before widespread use. Their implementation must be verified against actual local health data through public health validation checks.

In summary, more testing and adjustments for regional differences are critical to make these models adaptable across areas and set practical guidelines for real-world use in health care systems.

## Supplementary material

10.2196/74423 Multimedia Appendix 1 Prompts.

## Abbreviations

CNIC: Chinese National Influenza Center
ILI: influenza-like illness
LLM: large language model
LSTM: long short-term memory
MAE: mean absolute error
MAPE: mean absolute percentage error
MSE: mean squared error
N-BEATS: neural basis expansion analysis for time series
TFT: temporal fusion transformer
TiDE: time-series dense encoder
