# Supplementary material for: ChatGPT-Assisted Deep Learning Models for Influenza-Like Illness Prediction in Mainland China: Time Series Analysis
Source: J Med Internet Res. 2025 Jun 27;27:e74423. doi: 10.2196/74423 (PMC12227151; doi:10.2196/74423)
Supplement: Multimedia Appendix 1 [file jmir-v27-e74423-s001.docx]

I need a Python script to perform seasonal decomposition on a time series dataset and add marking lines to the plots. The specific requirements are:

1.Use 'xxx.csv' as the data source, which contains 'Time' and '%' columns.

2.Extract week and year information from the 'Time' column.

3.Convert the data into a time series and conduct seasonal decomposition.

4.Plot the decomposition with Observed, Trend, Seasonal, and Residual components.

5.Add vertical lines to indicate the start of each year and the midpoint (week 26) in each subplot.

Please provide a Python script for time series forecasting using the Darts library and a Transformer model. The script should include complete steps for data loading, preprocessing, model definition, training, prediction, and evaluation. Specific requirements are as follows:

1.Use pandas to load data from a CSV file. The data should have 'Year' and 'Week' columns, which need to be converted to a datetime index.

2.Preprocess the data:

(1)Convert 'Year' and 'Week' to a datetime index using the format '%G-W%V-%u'

(2)Set the frequency to weekly, starting on Mondays

(3)Use the '%' column as the target variable

(4)Apply appropriate scaling to the data

3.Implement data splitting:

(1)Use data from 2013 to 2023 as the training set

(2)Use data from 2024 as the test set

4.Use Darts' TransformerModel for modeling:

(1)Set appropriate input and output chunk lengths (e.g., 52 weeks for yearly patterns)

(2)Configure other hyperparameters as needed

5.Perform model training and prediction

6.Calculate and print evaluation metrics such as MAE, MSE, and MAPE

7.Use matplotlib to plot a comparison of predicted results and actual values

8.Include random seed setting to ensure reproducibility

9.Add appropriate comments explaining code functionality

The script should be well-structured, with separate functions for data loading, preprocessing, model training, prediction, and evaluation. Ensure that the code is efficient and follows best practices for time series forecasting with the Darts library.

"I'm encountering a SyntaxError: illegal target for annotation in my Python code when using type hints. The error occurs at line 91 where I wrote: "monitor": "train_loss", Python version: 3.11 What are the valid syntax rules for type annotations in this context, and how can I rewrite this code to comply with PEP standards while maintaining the intended type checking functionality? Please provide specific corrected code examples."
